# NK/T-cell lymphoma in a renal transplant recipient and review of literature

**DOI:** 10.4103/0971-4065.78078

**Published:** 2011

**Authors:** A. Mohapatra, A. Viswabandya, R. Samuel, A. N. Deepti, S. Madhivanan, G. T. John

**Affiliations:** Department of Nephrology, Christian Medical College, Vellore, India; 1 Department of Haematology, Christian Medical College, Vellore, India; 2 Department of General Pathology, Christian Medical College, Vellore, India

**Keywords:** NK/T-cell lymphoma, PTLD, renal transplantation

## Abstract

T-cell lymphomas, particularly NK/T-cell lymphomas are rare post transplantation malignancies. Only a few cases have been described. These tumors behave aggressively and the outcome is poor. We present here a case of NK/T-cell lymphoma who presented to us with an orbital swelling 9 years after renal transplantation, along with the review of literature. To the best of our knowledge, this is the first case of NK/T-cell lymphoma post-renal transplantation reported from India.

## Introduction

Post transplant lymphoproliferative disorders (PTLDs) are clinically and morphologically heterogeneous entities. These are EBV associated.[1] T-cell-derived PTLDs postrenal transplantation are rare and so far only a few cases of NK/T-cell-derived PTLD have been described in literature.[2–7]

We describe here a case of NK-cell PTLD in a patient after 9 years of transplantation.

## Case Report

A 50-year-old man from West Bengal with chronic glomerulonephritis underwent a pre-emptive renal transplantation from his mother in 1998 and was on triple immunosuppressant with prednisolone, cyclosporine, and azathioprine. He developed diabetes mellitus 5 months after the transplantation. Cyclosporine was withdrawn 1 year after the transplantation. There were no rejection episodes or major infections. He had normal graft function and was receiving prednisolone 10 mg/day and azathioprine 75 mg/day.

He presented to us in 2007 with gradual onset protrusion of the left eye with pain, redness, and diminished vision for 12 days. He did not have any focal neurological deficits, headache, or vomiting. There was no history of fever or weight loss.

**Figure 1:**
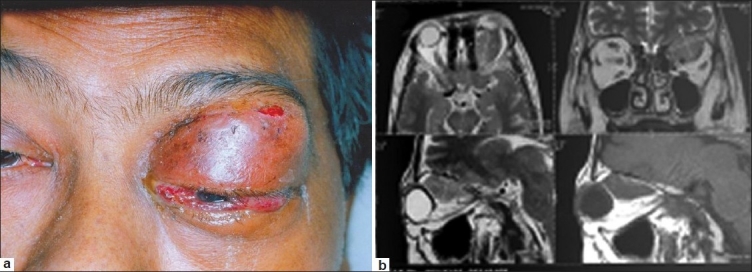
(a) Orbital mass at presentation (b) MRI findings

His weight was 56 kg and blood pressure was 120/80 mm of Hg. He was afebrile and did not have pallor, icterus, or lymphadenopathy. He had proptosis of the left eye with ocular redness and chemosis with gross lid edema. The right eye was normal [Figure 1a]. He had no focal neurological deficits. He had no neck stiffness or other meningeal signs.

Detailed ophthalmological evaluation revealed intact perception of light in left eye with inaccurate projection of light, restricted ocular movement 360°, and hemorrhagic chemosis. The left eye intraocular pressure was high (+24 mmHg). The disc margins were blurred with ischemia of the superior nasal quadrant of the retina with chorioretinal folds at the macula. The globe was indented superiorly.

MRI of brain showed a well-defined lesion with mildly irregular margins in the superior aspect of the left orbit, measuring ~2.5 × 1.6 × 2 cm, which was isointense on T1 and T2W images. The lesion was predominantly extraconal with a small intraconal extension and it was seen to abut the left optic nerve and partly encase it. The lesion had displaced the superior rectus levator palpebrae complex laterally and superior oblique medially with some involvement of the superior oblique muscle. There were few suspicious areas of bony deficiency in the superior margin of the orbit. A few long TR hyperintensities were noted in the cerebral white matter suggesting small vessel disease. The rest of the brain parenchyma was of normal signal intensity [Figure 1b]. Radiologists considered possibilities of either lymphoma or metastasis or pseudotumor of orbit.

**Figure 2:**
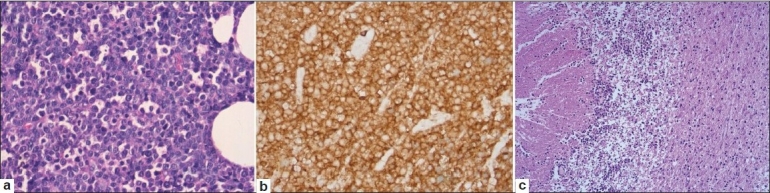
(a,b) Atypical lymphoid cells with negative staining for CD20; (c) Postmortem appearance of brain showing atypical lymphoid infiltrate

He underwent excision biopsy of the orbital mass the histopathology of which showed a diffuse infiltrate with medium sized lymphoid cells with scanty cytoplasm, round to indented nuclei, with granular chromatin, and inconspicuous nucleoli. The neoplastic cells were diffusely positive for CD3 and CD56 with an MIB-1 proliferation index of 85%. CD20, TdT, and EBV LMP – 1 were negative confirming the diagnosis to be T-cell lymphoproliferative disorder consistent with NK/T-cell lymphoma [Figure 2a and b].

His investigations showed Hb of 11 g/dl, total WBC count of 4500 with a normal differential count, and platelets of 151,000/cmm. His creatinine was 1.1 mg/dl, LDH was 480 U/L, uric acid was 3.5 mg/dl. His liver functions were within normal limit. His ultrasound of abdomen and chest X-rays were normal. Serum β2 microglobulin was not done. Viral serology for HbSAg, HCV, and HIV were negative. His bone marrow was normal without any evidence of involvement by lymphoma. His cerebrospinal fluid analysis showed less than five cells without any abnormal morphology, and CSF sugar and protein were within normal limit.

His immunosuppressive drugs were gradually tapered and he was treated with CHOP (cyclophosphamide, adriamycin, vincristine, and prednisolone) chemotherapy. He developed febrile neutropenia postchemotherapy. Although he was supported with broad-spectrum antibiotics and colony stimulating factor support, subsequently he developed sudden, rapid deterioration of sensorium. CT scan of brain showed a hypodense lesion in the left temporoparietal region and in the cerebellum. He also had hydrocephalus of the lateral and the third ventricle. Lymphomatous deposits or infection were considered, but CSF analysis done was normal. He was shifted to ICU as his sensorium continued to deteriorate. MRI was deferred in view of the poor general condition. Despite an external ventricular drainage, the patient deteriorated and died.

The histology of the brain showed moderate infiltrates of lymphoid cells with apoptosis, focal necrosis, and karyorrhectic debris predominantly in the cerebellar hemispheres involving the molecular and internal granular layers with focal extension into the underlying white matter [Figure 2c]. A similar, but relatively mild infiltrate of atypical lymphoid cells was also present in the midbrain, medulla, cervical cord, parietal, temporal, and occipital cortices with perivascular localization involving the parenchymal and leptomeningeal vessels.

## Discussion

Majority of PTLDs are of non-Hodgkin’s lymphoma subtype and makeup about 93% of lymphoma encountered in this setting. Out of those 86% of post-transplant lymphomas are of B-cell origin and about 14% of lymphomas are of T-cell origin, whereas less than 1% are of null cell origin.[8] The majority of early PTLDs are EBV-related. Our center data of PTLD on 27 patients showed that three were of T-cell lineage and no were NK/T-cell subtype.[9]

In the revised European–American lymphoma classification scheme, NK-cell lymphomas/leukemias are considered as a provisional entity, while in World Health Organization classification scheme, these tumors are classified as nasal-type extranodal NK/T-cell lymphoma and NK-cell leukemia. These are characterized by neoplastic cells of large granular lymphocyte morphology, the presence of cytoplasmic CD3 and CD 56 positivity, and a TCR gene arrangement in germ-line configuration.[10] Clinically, they are aggressive tumors with a poor prognosis.[11]

Our literature search showed only few cases of postrenal transplantation NK/T-cell lymphoma so far. Three of them were young and had presented at nasal cavity and cervical area and CNS each, and all of them were EBV positive and showed a good response to chemotherapy. Another case was a middle aged man who developed NK/T-cell lymphoma in bone marrow, which progressed rapidly to leukemia and he was also EBV positive. In another patient, lymphoma occurred 8 years after transplantation and it was negative for EBV markers. Table 1 denotes characteristics of a few cases of NK/T-cell lymphoma in postrenal transplant settings. Our case is the first case of NK/T-cell lymphoma postrenal transplantation reported in literature from India.

**Table 1:** NK/T-cell lymphoproliferative disorders postrenal transplant – A review of literature

Ref.	Sex	Age	Site	Histology	EBV
[2]	M	27	Nasal	NK/T	Positive
[2]	M	35	Nodal	NK/T	Positive
[7]	M	30	CNS	NK/T	Positive
[4]	M	43	Blood (leukemic phase) NK/T	Positive	
[5]	M	31	CNS	NK/T	Positive
[6]	M	27	Nasal	NK/T	Positive

NK-cell PTLD is extremely rare and they normally present in advanced leukemic phase. Our patient presented 9 years after renal transplantation and presented in an unusual site. The morphology was like large granular lymphocytic type with cytoplasmic CD3 being positive. The proliferation index was high with CD56 being diffusely positive, thereby confirming the NK subtypes. EBV LMP-1 (immunohistochemistry) done on tissue and EBV PCR done on peripheral blood were negative.

This patient had CD3 and CD56 positivity with a high proliferative index. Although T-cell rearrangement and TIA-1 were not evaluated, the morphology of cells with granularity and strong CD3 and CD56 expression suggest this to be a NK/T-cell lymphoma. Our patient also had a high proliferative index showing this to be an aggressive disease. We had also considered a possibility of peripheral T-cell lymphoma – not otherwise specified (PTCL–NOS) of WHO category in this patient – but these are usually CD56 negative. Although majority of NK/T-cell lymphomas are EBV positive, there are case reports of EBV negativity also described in literature as cited above.

Majority of patients with NK/T-cell PTLDs do poorly in spite of aggressive treatment. Immunosuppressive drugs were rapidly tapered for this patient and he was started on aggressive chemotherapy. In conclusion, even if T-cell PTLDs are rare, this should be considered a possibility in those patients who present late after transplantation with an extra nodal growth.
